# Screening method for *Staphylococcus aureus* identification in subclinical bovine mastitis from dairy farms

**DOI:** 10.14202/vetworld.2017.721-726

**Published:** 2017-07-01

**Authors:** Natapol Pumipuntu, Suphang Kulpeanprasit, Sirijan Santajit, Witawat Tunyong, Thida Kong-ngoen, Woranich Hinthong, Nitaya Indrawattana

**Affiliations:** 1 Department of Microbiology and Immunology, Faculty of Tropical Medicine, Mahidol University, Bangkok, Thailand; 2 Faculty of Medicine and Allied Health, HRH Princess Chulabhorn College of Medical Science Bangkok, Thailand

**Keywords:** bovine mastitis, deoxyribonuclease test, mannitol fermentation test, screening methods, *Staphylococcus aureus*

## Abstract

**Background:**

*Staphylococcus aureus* is one of the most important contagious bacteria causing subclinical bovine mastitis. This bacterial infection is commonly identified by determine the pathogen in bovine milk samples through conventional technique including coagulase test. However, this test has several disadvantages as low sensitivity, risk of biohazard, cost expensive, and limited preparation especially in local area.

**Aim:**

Aim of this study was to compare and assess the screening method, Mannitol fermentation test (Mannitol salt agar [MSA]), and deoxyribonuclease (DNase) test, for *S. aureus* identification in milk samples.

**Materials and Methods:**

A total of 224 subclinical bovine mastitis milk samples were collected from four provinces of Thailand and determined *S. aureus* using conventional method and also subjected to the screening test, MSA and DNase test. The sensitivity, specificity, positive predictive value (PPV), and negative predictive value (NPV) among both tests were analyzed and compared to the tube coagulase test (TCT), as reference method. Immunological test by latex agglutination and molecular assay by determined *spa* gene were also used to identify and differentiate *S. aureus*.

**Results:**

A total of 130 staphylococci were isolated by selective media, Gram-stain, and catalase test. The number of *S. aureus* which identified using TCT, MSA and DNase test were 32, 102, and 74 isolates, respectively. All TCT results were correlated to results of latex agglutination and *spa* gene which were 32 *S. aureus*. MSA showed 100% sensitivity, 28.57% specificity, 31.37% PPV, and 100% NPV, whereas DNase showed 53.13% sensitivity, 41.84% specificity, 22.97% PPV, and 73.21% NPV. DNase test showed higher specificity value than MSA but the test presented 26.79% false negative results whereas no false-negative result from MSA when comparing to TCT.

**Conclusion:**

MSA had a tendency to be a good preference for screening *S. aureus* because of its high sensitivity and NPV. The result from this study will improve a choice to use a screening test to diagnose *S. aureus* of veterinary field for prompt disease controlling and effective treatment.

## Introduction

Bovine mastitis is a disease of the most prevalence and costly diseases in dairy cows or some livestock milk industries with losses lead to reducing of milk production, changing in milk composition, discarding or low quality milk, increasing veterinary services, and increasing labor costs [1]. This inflammation of the mammary gland can be divided into two types that manifested by the appearance of inflammation at the udder of dairy cow; asymptomatic (subclinical mastitis) and symptomatic mastitis (clinical mastitis), whereas subclinical mastitis can be occurred up to 40 times more common than clinical cases [2]. Thus, this subclinical mastitis seems to be an important type of mastitis in dairy cow because it is a hidden mastitis or invisible problem in the herd [3] and needed to be more concern. *Staphylococcus* species are one of the main etiological bacteria that cause bovine mastitis especially for *Staphylococcus aureus* which is considered as a contagious pathogen that often cause bovine mastitis [4]. These bacteria can spread from infected cow to another cow in a herd by mainly through many routes as contaminated milking equipment or through hands of farmers. *S*. *aureus* infection in subclinical bovine mastitis becomes to be a large and important problem in dairy farm industry [5]. There are estimates that about 80-100% of all dairy herds have at least some *S. aureus* mastitis, with from 5% to 10% of infected cows [6]. In Thailand, the prevalence of *S. aureus* infection in dairy cattle subclinical mastitis is also different in each study site such as 8% and 3% of the bacterial isolates in Chiang Mai [7] and Khon Kaen [8], respectively.

Conventional method is the gold standard for identification of staphylococcal infection from the clinical specimen. Tube coagulase test (TCT) is the one of biochemical tests that commonly used to differentiate *S. aureus* from coagulase-negative staphylococci for a reason that the bacteria can produce coagulase protein to promote coagulation [9-12]. This protein enzyme can activate the nonproteolytic activation of prothrombin and cleavage of fibrinogen [13]. In routine laboratory, TCT is normally prepared from plasma of human or rabbit or horse [14]. Nevertheless, the differentiation of *S. aureus* by TCT spent a times to monitor and interpret the results [15]. Moreover, the interpretation of TCT needs the expertise to clarify the results [16]. Risk of biohazard from TCT can be happen when the plasma derived from the infectious plasma whatever from human or animal [17]. Furthermore, some *S. aureus* may produce low amount of coagulase which render false negative result in TCT [18].

Beside the disadvantage of TCT, Mannitol salt agar (MSA) and deoxyribonuclease (DNase) testing are the other biochemical methods which can be used for screening the differentiation of *S. aureus* from other species. Due to *S. aureus* can ferment mannitol sugar and produces an acid at the end product when inoculated on MSA result in the phenol red indicator change the color from red to yellow [19]. Since *S. aureus* has an ability to produce enzyme DNase which can hydrolyze nucleic acid in DNase medium agar, then it will be seen a clear zone around bacterial colonies [20]. From the limitation in some laboratories which in short supply to find some plasma to perform the coagulase test by the reason of its expensive and risk of biohazard that we mentioned above. MSA and DNase are interested to use instead of TCT [21]. Therefore, this study aims to evaluate the efficiency of MSA and DNase for screening *S. aureus* in milks of subclinical bovine mastitis cases from four provinces in Thailand comparing to TCT. The sensitivity, specificity, positive predictive value (PPV), and negative predictive value (NPV) were determined. The result from this study will provide a choice of a screening test for diagnose *S. aureus* of veterinary field for further prompt disease controlling and effective treatment.

## Materials and Methods

### Ethical approval

All procedures performed in this study were approved by the Faculty of Tropical Medicine-Animal Care and Use Committee, Mahidol University, Thailand (protocol no. 002-2016).

### Sample collection and preparation

Individual 224 milk samples were aseptically collected from subclinical bovine mastitis cases in from 52 dairy farms which 8 different areas Thailand, i.e.; Saraburi, Lopburi, Nakorn Ratchasima, and Maha Sarakham provinces during September 2015 to April 2016. All samples were inoculated on Columbia blood agar supplemented with nalidixic acid and colistin sulfate for *Staphylococcus* spp. and *Streptococcus* spp. (Oxoid, Hampshire, UK) and incubated at 35°C for 18 h. The suspected bacterial colonies were subjected to conventional methods such as Gram-stain, catalase test, coagulase test, MSA, and DNase.

### Screening test for S. aureus

Staphylococcal isolates which showed positive result for catalase test were subcultured on human blood agar and incubated at 37°C for 24 h. Then, the single colonies were subjected to the screening test, i.e., MSA, DNase and TCT, respectively.

### MSA

**Figure-1:**
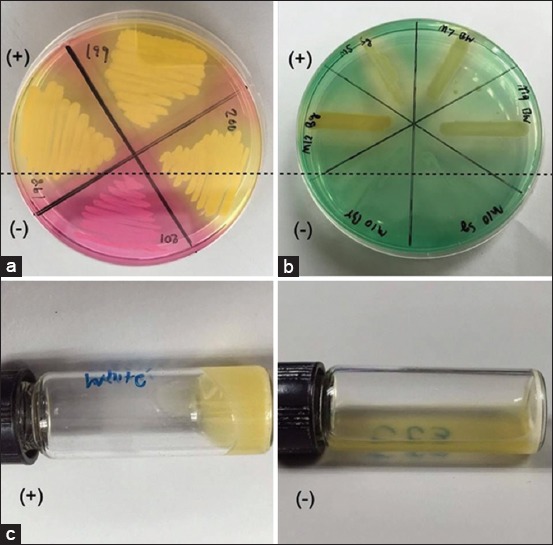
Screening tests for identification of *Staphylococcus aureus* from subclinical bovine mastitis milk sample. (a) Mannital salt agar; (b) deoxyribonuclease test and (c) tube coagulase test. (+) - positive result; (−) - negative result.

MSA contains 1% mannitol, 7.5% sodium chloride, phenol red indicator, and peptone. *S. aureus* can tolerate and survive in high salt condition in this medium and can grow on it whereas the other bacteria will be inhibited by the high salt concentration from the media. *S. aureus* can ferment the sugar mannitol and from this ability it produces an acid at the end product that changes phenol red indicator into yellow. In this experiment, the suspected single bacterial colony was inoculated on MSA plate (Oxoid, Basingstoke, UK), incubated at 35°C for 24 h and observed the indicator change (Figure-1).

### DNase test

DNase agar contains 2% tryptose, 0.2% deoxyribonucleic acid, 0.5% sodium chloride, and methyl green indicator. *S. aureus* has ability to produce enzyme DNase which can hydrolyze nucleic acid in DNase medium and was seen a colorless zone around their colonies. In this study, the single bacterial colony was inoculated on DNase plate (Oxoid, Basingstoke, UK), incubated at 37°C for 18 h and observed the colorless zone around bacterial colony which indicated the colony of *S. aureus* (Figure-1).

### TCT

TCT is prepared using human or animal plasma. The test aspired for detecting free coagulase of *S. aureus*. Free coagulase is secreted from extracellular specifically by *S. aureus* that reacted with the coagulase reacting factor in plasma that able to from a complex together, which called thrombin. The thrombin has ability to convert fibrinogen to fibrin resulting in clotting of plasma. This thrombin can be called staphylothrombin. In this study, TCT was done by adapted the protocol from Murray, 2003 [22]. The full loop of fresh suspected colonies with 1 ml of human plasma, then incubated the mixture at 37°C and observed for clotting formation by gently tilting it horizontal from the vertical after incubated for 4, 6 and 24 h. Plasma clotting was considered to be a positive result for coagulase test (Figure-1).

All methods have subjected with *S. aureus* ATCC 25923 as positive control and *Staphylococcus epidermidis* ATCC 12228 was used as a negative control, respectively.

### Verify assays for differentiate S. aureus

#### Latex agglutination

The suspected bacterial isolates, which showed gram positive cocci and positive for catalase test, were determined to be *S. aureus* by Staphaurex Plus kit (Remel Europe Ltd., Dartford, UK) based on immuno-agglutination technique. The latex particle was coated with rabbit immunoglobulin G (IgG) and fibrinogen which can render the interaction of fibrinogen and *S. aureus* clumping factor, the Fc portion of IgG and *S. aureus* protein A and specific IgG and *S. aureus* cell surface antigens. The experiment was done following to the manufacture’s protocol [23]. Two drops of bacterial suspension was mixed with a drop of latex particle reagent on a reaction card. Then, the card was rotated smoothly for 30 seconds before observing noticeable agglutination. *S. aureus* strain ATCC 25923 was used as positive control which showed the mixture agglutination whereas no agglutination when tested with *S. epidermidis* strain ATCC 12228, a negative control. The staphaureux-positive isolates were determined the *spa* gene by polymerase chain reaction (PCR).

#### Amplification of spa gene

**Figure-2:**
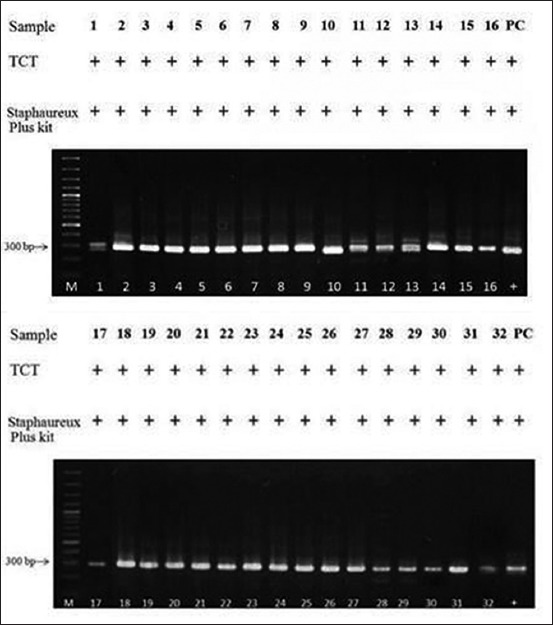
*Staphylococcus aureus* identification by using tube coagulase test, Staphareux Plus kit and *spa* gene amplification. Lane M, 100 bp DNA ladder; Lane 1-32, Staphylococal isolate DNA samples; Lane PC, *S. aureus* ATCC 25923 (positive control). *Spa* amplicon is 300 bp. (+) - positive result.

Several studies used the molecular method to determine *S. aureus* by amplify the *spa* gene that is encoded for protein A [24,25]. Bacterial genomic DNA was extracted using DNA extraction kit (Geneaid, Taiwan). The PCR mixture (25 μL) consisted of 1 μM of forward primer (5’-CAAGCACCAAAAGAGGAA-3’) and reverse primer (5’-CACCAGGTTTAACGACAT-3’), 100 ng of DNA template, 2.5 μL of 10×Taq PCR buffer, 0.2 mM dNTP, 2 mM MgCl_2_, and 1 unit of Taq DNA polymerase (Thermo Scientific, USA). The PCR mixture was subjected to the following thermal cycle conditions using the T100^™^ Thermal Cycler (BioRad, USA): 95 min of 95°C before 35 cycles of amplification at 95°C for 45 s, 58°C for 45 s, and 72°C for 45 s, followed by a final extension at 72°C for 10 min. The PCR products were analyzed by 1.5% agarose gel electrophoresis and ethidium bromide staining [26]. The DNA bands, size 300 base pairs (Figure-2), were observed under a Gel Doc™ XR+ System (Biorad, USA). *S. aureus* ATCC 25923 and *S. epidermidis* ATCC 12228 were used in this study as positive and negative control, respectively.

### Efficiency analysis

The SPSS software (version 20.0) was used for statistical analysis. The sensitivity and specificity of MSA and DNase were analyzed using two × two tables as the followings formulas:


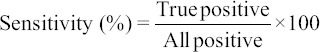



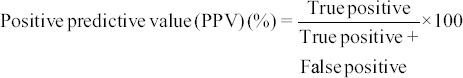



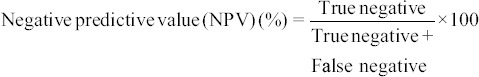


## Results

From total 224 milk samples, 130 *Staphylococcus* spp. were isolated by the selective media (Columbia supplemented with the inhibitors), Gram-stain and catalase test. As showed in Table-1, there were 78.46 % (102/130), 56.92% (74/130), and 24.65% (32/130) staphylococcal isolates showed positive result on MSA test, DNase test and TCT test, respectively. All 32-positive TCT isolates were also positive for both Staphaureux Plus and *spa* gene as showed in Figure-2.

**Table-1:** Screening for *Staphylococcus aureus* from subclinical bovine mastitis milk sample by MSA and DNase test comparing to tube coagulase test.

Biochemical test	Number isolates (n)	Tube coagulase test	

		Positive (n)	Negative (n)
MSA (+)	102	32	70
MSA (−)	28	0	28
DNase (+)	74	17	57
DNase (−)	56	15	41
MSA, DNase (+,+)	55	17	38
MSA, DNase (+,−)	47	16	31
MSA, DNase (−,+)	20	0	20
MSA, DNase (−,−)	8	0	8

Total 130 Staphylococcal isolates. MSA=Mannitol salt agar, DNase=Deoxyribonuclease test

The efficiency of MSA showed 100% sensitivity, 28.57% specificity, 31.37% PPV, and 100% NPV. Whereas, the efficiency of DNase showed 53.13% sensitivity, 41.84% specificity, 22.97% PPV, and 73.21% NPV (Table-2). DNase test showed higher specificity value than MSA but the test presented 26.79% (15/56) false negative results whereas no false-negative result from MSA when comparing to TCT. The efficiency of the combination tests, MSA and DNase, for screening *S. aureus* also determined (Table-2). The combined test presented 100% sensitivity, 17.39% specificity, 30.9% PPV, and 100% NPV.

**Table-2:** The calculated efficiency of MSA and DNase tests for screening of *Staphylococcus aureus* from subclinical bovine mastitis milk sample.

Biochemical tests	Sensitivity (%)	Specificity (%)	PPV (%)	NPV (%)
MSA	100	28.57	31.37	100
DNase	53.13	41.84	22.97	73.21
MSA and DNase*	100	19.15	30.9	100

* Only MSA, DNase (+,+) and MSA, DNase (−,−) were considered for this calculation. MSA=Mannitol salt agar, DNase=Deoxyribonuclease test, PPV=Positive predictive value, NPV=Negative predictive value

## Discussion

Screening method for identification of *S. aureus* is important and necessary for prevention and control in dairy herd for veterinary field and animal health. There has less report about the evaluation of screening method for this pathogen especially with clinical sample from animal. Our study demonstrates that MSA show high sensitivity and can be used as screening test for differentiates *S. aureus* from animal specimen which possible infected with various species of *Staphylococcus*. The results similar to other reported from Kateete *et al*. [27] which screening clinical staphylococcal isolates from human by using MSA and combination method (MSA/DNase). They reported that MSA had higher sensitivity and NPV but lower specificity and PPV than DNase test. They also concluded that a combination of MSA and DNase which had highest sensitivity was the best choice for screening and identifying of *S. aureus* clinical isolates [27]. However, our study showed similar sensitivity value (100%) of MSA and the combination method (MSA and DNase test). Moreover, screening *S. aureus* from *Staphylococcus* isolates using MSA method showed higher specificity than the combination test. Consequently, MSA would be the best choice to screen and identify *S. aureus* isolated from bovine mastitis milk rather than the combined method even the PPV is very close (31.4% vs. 30.9%).While DNase test, presented low sensitivity and specificity, may not appropriate to use for screening and identifying as a single test. Furthermore, MSA had 100% NPV, it means that negative-MSA result can reliable as true negative result. MSA is appropriate to rule out the negative result as non-*S. aureus* isolates. Besides, many studies reported that DNase test showed lower sensitivity than MSA when compare the results with TCT [17,27,28]. Although DNase test showed higher sensitivity value than TCT, it may not suitable to use for screening *S. aureus* infection in dairy cows. Because of the DNase test showed high false negative results which render the infected cow (false negative-DNase test) will not treat and still be with the uninfected animal. Thus, the mastitis infection can disseminate in the dairy farm via this management. Therefore, it is extremely important to choose the appropriate screening test with provide the true negative result for quarantine *S. aureus* infected animal out from uninfected animal in order to medicate, prevent and limit the exposure and expansion of the infection in farms.

Furthermore, if we compared MSA, DNase and TCT in the terms of economic or cost of commercial media, they were found that MSA was very low priced. It was cheaper than DNase test 1.5 times and cheaper than TCT about 50 times. Therefore, MSA is a good choice to be an optimal choice for initial screening of *S. aureus* infection in dairy herd.

## Conclusion

The use of MSA seems to be a valuable tool for initial screening and identifying of *S. aureus* clinical isolates in bovine milk sample. It can be used to screen *S. aureus* infection in local laboratories nearby agricultural fields in order to assist veterinarians for diagnosing and making a decision to readily medicate, control and prevention. However, if veterinarians suggest culling the infected cows out of the farm as *S. aureus* infection after MSA positive result, they need to be confirmed this bacterial infection by TCT or other molecular techniques before culling.

## Authors’ Contributions

NP and NI planed and designed of the study. The samples were collected in the fields by NP. Laboratory works were done by NP, SK, SS, WT, TK and WH. NI and NP analyzed the data and achieved statistical analysis. NP drafted and revised the manuscript under the advice from NI. Finally, all authors read and approved the final manuscript.
